# Identifying factors of psychological distress on the experience of pain and symptom management among cancer patients

**DOI:** 10.1186/s40359-016-0160-1

**Published:** 2016-11-02

**Authors:** Tamara A. Baker, Jessica L. Krok-Schoen, Susan C. McMillan

**Affiliations:** 1 Department of Psychology, University of Kansas, 426 Fraser Hall, 1415 Jayhawk Blvd, Lawrence, KS 66045 USA; 2 Comprehensive Cancer Center, The Ohio State University, 1590 N High St., Suite 525, Columbus, OH 43210 USA; 3 University of South Florida, College of Nursing, 12901 Bruce B. Downs Blvd, MDC Box 22, Tampa, FL 33612 USA

**Keywords:** Pain frequency, Pain presence, Psychological distress, Physical functioning

## Abstract

**Background:**

Epidemiological evidence suggests the impact psychological distress has on symptomatic outcomes (pain) among cancer patients. While studies have examined distress across various medical illnesses, few have examined the relationship of psychological distress and pain among patients diagnosed with cancer. This study aimed to examine the impact psychological distress-related symptoms has on pain frequency, presence of pain, and pain-related distress among oncology patients.

**Methods:**

Data were collected from a sample of White and Black adults (*N* = 232) receiving outpatient services from a comprehensive cancer center. Participants were surveyed on questions assessing psychological distress (i.e., worry, feeling sad, difficulty sleeping), and health (pain presence, pain frequency, comorbidities, physical functioning), behavioral (pain-related distress), and demographic characteristics.

**Results:**

Patients reporting functional limitations were more likely to report pain. Specifically, those reporting difficulty sleeping and feeling irritable were similarly likely to report pain. Data further showed age and feeling irritable as significant indicators of pain-related distress, with younger adults reporting more distress.

**Conclusions:**

It must be recognized that psychological distress and experiences of pain frequency are contingent upon a myriad of factors that are not exclusive, but rather coexisting determinants of health. Further assessment of identified predictors such as age, race, socioeconomic status, and other physical and behavioral indicators are necessary, thus allowing for an expansive understanding of the daily challenges and concerns of individuals diagnosed with cancer, while providing the resources for clinicians, researchers, and policy makers to better meet the needs of this patient population.

## Background

Despite advances in supportive cancer care, psychological distress remains as a significant issue among individuals diagnosed with cancer [1, 2]. The level of psychological and emotional distress associated with a cancer diagnosis contributes to increased rates of co-morbidities and mortality, while reducing quality of life and adherence to medical treatment [3, 4]. The magnitude of distress is often concomitant with the diagnosis, treatment and symptoms associated with the chronic illness [4].

### Psychological distress and a cancer diagnosis

The diagnosis of cancer and the uncertainty of treatment (and a cure) may evoke emotional discontent and related psychological distress. The innumerable demands placed on the patient puts them at a more vulnerable mental and physical state, thus experiencing more psychological distress and distress-related symptoms. [5] While psychological distress may be all encompassing of the multiple demands, experiences, and feelings of those diagnosed with cancer, it remains as a source of inquiry in understanding the influence it has among certain identified characteristics [5, 6]. Studies show that distress dominates across a continuum of cancer types (breast, lymphoma) [6–11] and related symptoms (sleep, fatigue, pain) [12, 13].

### Pain and psychological distress

Evidence contends a complex interaction of pain and psychological distress among patients diagnosed with cancer in general [10, 13]. Yet, with more advanced stages of cancer, this dynamic relationship has shown to impact cognition, personality, and behavior; and evoke emotional disturbances such as depression and anxiety [10, 12, 13]. Yet, the multi-dimensionality of the pain and psychological distress dyad addresses something more complicated than the diagnoses and related symptoms. Because of the nature of pain and distress-related symptoms (e.g., depression, anxiety, worry), there is contradictory evidence suggesting whether the pain experience precedes or is the result of a psychological condition/symptoms. [10, 14] This is all the more important when assessing the dynamics of pain and psychological distress among patients diagnosed with cancer. With the emotional toll of a cancer diagnosis, it is critical that health care professionals consider the complexity of issues associated with the disease and how this relationship is contingent on a myriad of cultural, behavioral, physical, and social factors that are not exclusive, but rather coexisting determinants of health [14–20].

Despite documenting the relationship between psychological distress and symptoms commonly associated with disease progression; identifying the influence of psychological distress and related symptoms on the pain experience has not been thoroughly examined among adults diagnosed with cancer. Guided by the concepts of the biopsychosocial (BPS) theoretical approach, which provides a general model conjecturing the multidimensionality of health, with the amalgamation of biological, psychological and social factors contributing to the context of health and illness [21, 22], this study examined the influence and association of identified psychological distress-related symptoms (worry, feeling sad, difficulty sleeping, difficulty concentrating, feeling nervous, feeling irritable), and health (comorbidities, physical functioning) and demographic characteristics as determinant indicators of pain frequency, presence of pain, and pain-related distress. To contribute to our understanding of these relationships, this study specifically aimed to: (1) describe the frequency of identified psychological-distress related indicators, (2) examine factors associated with pain-related distress, pain frequency, and pain presence, (3) determine the amount of unique variance in pain frequency and pain-related distress accounted for by specific health variables, while controlling for demographic and psychological-distress related symptoms (independently and collectively), and (4) predict the pattern of pain presence in a sample of patients diagnosed with cancer.

## Method

### Participants

Data were taken from a parent project designed to examine pain, adherence to pain medication, and constipation among patients receiving outpatient services from a National Cancer Institute (NCI)-Designated Comprehensive Cancer Center. To be included for study participation patients had to self-identify as non-Hispanic White or Black; ≥ 18 years of age; have a cancer diagnosis at any stage; currently receiving cancer treatment (i.e., radiation, chemotherapy, or combination); be cognitively intact; and able to provide written informed consent to participation. Patients who enrolled in a cancer pain intervention or non-pharmacologic intervention within the past year, or unable to read and understand English, were not eligible to participate in the project. This investigation was approved by the university’s Institutional Review Board and the cancer center’s Protocol Review Monitoring Committee.

### Procedure

Data were collected through chart reviews and patient interviews assessing specific psychological distress-related symptoms, pain, and health and demographic characteristics. Research Assistants were responsible for patient recruitment, interviews, and administering the questionnaire. All patients were approached by a Research Assistant during the patient’s medical visit (either in the waiting area, while being triaged, or receiving treatment) to determine their interest and eligibility for study participation. Upon providing consent, each interview (and survey) lasted approximately 30 min and was conducted in a private area in the clinic.

### Measures

#### Dependent variable

##### Pain (frequency, presence, and related-distress)

Pain frequency, presence, and pain related-distress were assessed using the 32-item Memorial Symptom Assessment Scale (MSAS). The measure consists of two validated subscales: physical symptoms (PHYS) and psychological (PSYCH), that assess the frequency, presence, and distress related to each symptom. For purposes of this investigation, only the pain symptom from the PHYS subscale (frequency, presence, and distress scores) was included in subsequent analyses. The presence of pain was assessed as a dichotomous variable, with response choices as either yes or no (experiencing pain or not). Pain frequency was measured on a five-point Likert scale (0 = not at all to 4 = very severe), with a higher score endorsing more of the symptom. Pain-related distress was similarly rated on a five-point Likert scale (how much does the symptom distress or bother you; 0 = not at all to 4 = very much), with higher scores suggesting more distress resulting from pain. The MSAS has established validity and reliability among patients diagnosed with cancer and undergoing cancer treatment [23].

### Independent variables

#### Psychological symptoms

The MSAS-PSYCH subscale was used to measure the frequency, presence, and distress associated with six psychological symptoms (difficulty concentrating, feeling nervous, difficulty sleeping, feeling sad, worry, feeling irritable). Symptom frequency was measured on a five-point Likert scale (0 = not at all to 4 = very severe), with a higher score endorsing more of the symptom. Presence of the symptom was assessed as a dichotomous variable, with response choices as either yes or no (experiencing the symptom or not). Symptom-related distress was similarly rated on a five-point Likert scale (how much does the symptom distress or bother you; 0 = not at all to 4 = very much), with higher scores denoting more distress related to the symptom. Previous studies report strong reliability coefficients for the psychological subscale (α = 0.83-0.88) [23]. Scale analysis for this study revealed similar internal consistency for the PSYCH subscale (α = 0.73).

### Health variables

A series of single-item questions assessed the patient’s primary metastatic site, stage of disease, treatment stage (under treatment with curative, under treatment with palliative, or in remission), and cause of pain (cancer-related, non-cancer related or both). Level of performance (i.e., physical functioning) was measured using the Eastern Cooperative Oncology Group Performance Status (ECOG-PS). Response choices were rated on a five-point Likert scale, with higher scores suggesting complete disability (0 = fully active to 4 = completely disabled) [24, 25].

### Demographics characteristics

Five demographic variables were included in the analyses: age, sex, race/ethnicity, education, and marital status. Age was scored in a continuous format. Sex was treated as a dichotomous variable (male/female). Race was assessed via five nominal categories (White/Caucasian, Black/African American, Hispanic/Non-Caucasian, Asian/Pacific Islander, and other). Education was assessed as the total number of years of formal schooling. Marital status was scored as a dichotomous variable (divorced/widowed/single vs. married).

### Statistical analysis

Descriptive analyses were calculated to check for missing and outlying data, and to provide a profile of the sample’s demographic (age, race, gender, education, marital status), health (metastatic site, stage of disease, treatment stage, cause of pain, physical functioning), and pain (frequency, presence, related-distress) characteristics, and psychological symptoms (difficulty concentrating, feeling nervous, difficulty sleeping, feeling sad, worrying, feeling irritable). A series of Pearson Product-Moment correlation coefficients (pairwise deletion) were examined to assess the strength of the bivariate associations between pain frequency and each psychological symptom (PSYCH variables). A forward stepwise logistic regression model was calculated to determine significant predictors of pain presence (yes/no), with sex, race, education, age, marital status, physical functioning, and the six PSYCH variables entered as covariates in the final regression model. Separate hierarchical multiple regression models were similarly calculated to determine the amount of unique variance in pain frequency and related distress accounted for by specific health variables, while controlling for the demographic and psychological symptoms (independently and collectively).

The regression procedure entered the predictor variables in three models. Demographic variables (age, race, sex, education, marital status) were entered first (Model I), followed by physical functioning (ECOG) (Model II). The psychological symptoms (PSYCH variables) were entered as the final set of predictor variables (Model III). Standardized beta coefficients were reported to describe the relative importance of the predictor variables within the regression model. Statistical significance for all analyses were determined with the probability of a Type I error, *p* ≤ .05. All statistical analyses were performed with the Statistical Package for Social Sciences (SPSS Inc., Chicago, IL) version 22.0.

## Results

### Demographic, pain, and health characteristics

Data consisted of 232 adult patients, with a mean age of 55 (SD = 12.24) years and 13.64 (SD = 2.43) years of education. The majority of the sample were white (85 %), with an equal number of males (*n* = 116) and females (*n* = 116). Sixty-seven percent of the participants were married, with more than half residing with a spouse (60 %) and living in their own home (93 %). Lymphoma (23 %), lung (15 %), and breast (15 %) were the most common cancer diagnoses. Less than half of the sample (47 %) was diagnosed at a stage IV, with 21 % not knowing their diagnostic stage. Approximately 58 % of the patients reported their pain as cancer-related, with less than one-third reporting pain as a result of both cancer and a non-cancer medical condition(s). The sample reported an average of 2.48 ± 1.08 (0–4 Likert scale) on pain frequency, with a similar score of 2.60 ± 1.22 for related distress. Other demographic and health characteristics are provided in Table 1.

**Table 1:** Demographic, health, and pain characteristics (*N* = 232)

Variables	M ± SD/%
Age	55.6 ± 12.2
Female (n)	116
Education	13.64 ± 2.43
Marital Status (% married)	67 %
Pain due to cancer	58 %
Physical functioning (ECOG; able to do light housework)	59 %
Pain presence (yes/no)	75 %
Pain-related distress	2.60 ± 1.22
Pain frequency	2.48 ± 1.08

### Presence, frequency, and distress of psychological symptoms

#### Symptom presence

Table 2 shows that more than half of the participants reported difficulty sleeping and being worried, with another 45 % feeling irritable. Forty-one percent of the patients reported feeling sad and difficulty concentrating, with approximately one-third of the sample feeling nervous. Participants had an overall PSYCH symptom distress and frequency mean score of 1.71 SD = 1.23) and 1.61 (SD = 1.07), respectively.

**Table 2:** Prevalence of distress-related symptoms

PSYCH variables	Percent
Difficulty Concentrating	41
Feeling Nervous	31
Difficulty Sleeping	55
Feeling Sad	41
Worrying	52
Feeling Irritable	45

### Symptom frequency and distress

Difficulty sleeping (M = 2.32, SD = 1.08) and worry (M = 2.15, SD = 1.10) were reported as the most frequent psychological symptom, with difficulty concentrating (M = 1.74, SD = .92) and feeling sad (M = 1.85, SD = 1.02) as the least frequent. Similarly, difficulty sleeping (M = 2.50, SD = 1.22) and feeling nervous (M = 2.34, SD = 1.29) were the most psychologically distressing symptom, with difficulty concentrating (M = 1.99, SD = 1.41) and feeling irritable (M = 2.07, SD = 1.26) as the least distressing.

### Association of psychological symptoms and pain presence, frequency, and distress

The presence of pain was significantly associated with all six PSYCH variables: feeling nervous (*r* = .26, *p* < .001), feeling sad (*r* = .28, *p* < .001), worry (*r* = .32, *p* < .001), being irritated (*r* = .34, *p* < .001), difficulty sleeping (*r* = .19, *p* < .01), and concentrating (*r* = .30, *p* < .001). None of the six PSYCH variables were related to pain frequency. Results further showed a moderate relationship between patients who reported being distressed from their pain (bothered by their pain) and being irritated (*r* = .22, *p* < .01). None of the remaining psychological symptoms were associated with pain-related distress (Table 3).

**Table 3:** Association between pain (Presence and distress-related) and psychological variables

PSYCH variables	r
Pain presence	
Difficulty Concentrating	.30^**^
Feeling Nervous	.26^**^
Difficulty Sleeping	.19^*^
Feeling Sad	.28^**^
Worrying	.32^**^
Feeling Irritable	.34^**^
Pain-related Distress	
Feeling Irritable	.22^*^

^*^ *p* < .01; ^**^ *p* < .001

### Pattern of pain presence

Predictors of the presence of pain (yes/no) were calculated after controlling for demographic, health, and psychological covariates (i.e., age, race, sex, marital status, education, physical functioning), and the six psychological symptoms (difficulty concentrating, feeling nervous, difficulty sleeping, feeling sad, worrying, feeling irritable). Table 4 shows that younger patients (OR = .96, 95 % CI = .93 - .99 *p* < .05) were more likely to report pain than the older patients. It was similarly found that patients with more (physical) functional limitations (OR = 3.82, 95 % CI = 1.90 - 7.65; *p* < .001) were three times more likely to report pain. Analyses further showed that patients who reported difficulty sleeping (OR = 2.25, 95 % CI = 1.02 - 4.95; *p* < .05) and feeling irritable (OR = 2.95, 95 % CI = 1.14 - 7.62; *p* < .05) were similarly likely to report pain. None of the remaining demographic, pain or psychological symptoms were statistically significant indicators of pain presence.

**Table 4:** Indicators of pain presence

Variables	Odds ratio	*p-value*	95 % CI
Age	.96	<.05	.93–.99
Physical functioning	3.82	<.001	1.90–7.65
Difficulty sleeping	2.25	<.05	1.02–4.95
Feeling irritable	2.95	<.05	1.14–7.62

Variables initially tested: age, race, sex, education, marital status, physical functioning, pain presence, worry, difficulty sleeping, feeling sad, irritable, difficulty concentrating, feeling nervous

### Indicators of pain frequency and pain-related distress

Neither model examining the unique variance in pain frequency (F[12, 168] = 1.43, *p = NS*) and pain-related distress (F[12, 166] = 1.73, *p = NS*) was significant. Although the collective models were not significant, there were individual variables that contributed to the variance of each dependent variable (pain frequency and related distress).

For pain frequency as the outcome variable, the first step in model development involved entering the demographic variables (race, age, sex, education, and marital status; Model I), which accounted for 7 % of the total variance. Age (β = -.17, *p* < .05) was the only significant demographic predictor in the first model, with younger adults experiencing more pain. Physical functioning was entered in the second model (Model II), and was not a significant indicator of pain frequency. Age, however was retained as a significant predictor (β = -.19, *p* < .05) when entered in Model II. After controlling for the demographic and health indicators, none of the psychological symptoms (Model III) were significant indicators. The effect of age was the only variable that remained as a significant predictor of pain frequency, after controlling for all other variables.

Similarly, the pain-related distress model was not significant, however the final model showed that age (β = -.17, *p* < .05) and feeling irritable (β = .23, *p* < .01) were significant indicators of pain related-distress.

## Discussion

There is a continued need to understand the impact psychological distress and related symptoms have on the pain experience among those diagnosed with cancer. This study aimed to quantify the effects of identified distress-related symptoms on pain frequency, presence, and distress among Black and White patients receiving outpatient services at a comprehensive cancer center. Results showed interesting preliminary data on the effects of identified psychological symptoms on pain-related health outcomes.

With more than 75 % of the sample reporting pain, these results are significant in documenting the pain and psychological well-being dyad among cancer patients. The continued need to understand the multi-faceted approach to achieving optimal pain management, in addition to assessing the patient’s pain frequency and related distress, validates the complexity of a cancer diagnosis and how these symptoms (e.g., pain, depression, physical impairment) co-exist with one another [23].

While the Institute of Medicine (IOM) acknowledges pain as a disease in itself, it is similarly recognized as a serious outcome for a number of physically debilitating medical conditions. Dekker and colleagues [26] provide a cogent description of the path from disease to physical impairment, citing that avoidance of certain pain-related activities promote a self-reinforcing cycle of activity avoidance, pain and limited functional capacity. Several investigations show similar findings among cancer patients [27–30]. Despite the known benefits of physical activity, we must recognize some of the barriers a cancer diagnosis presents on a patient’s ability to perform certain physical everyday tasks. For example, asking a patient to walk one half of a mile each day (as a means of exercise) may be a serious challenge, particularly for those who may recently received treatment (e.g., radiation, surgery). Not only are there the physical demands of performing the task, but there are the emotional (e.g., depression) constraints that may impact one’s ability or willingness to perform the activity.

We similarly found that age was an important indicator of pain, with younger patients reporting more pain than their older counterparts. Our findings corroborate with prior research suggesting that the experience of pain and related psychological distress differs across age groups, with older cancer patients reporting less pain frequency, frequency of distress than younger patients [31–33]. This may be the result of the elderly patient having developed more effective coping mechanisms to deal with the burden and experience of pain [32]. There is also the notion that the elderly patient may have accepted the pain as part of the aging process. This, of course, is and should not be normative thinking, considering the number of elder adults who neither report nor experience pain; acute, chronic, or otherwise. Examining the pain experience among older cancer patients continues to be a growing public health concern that warrants further investigation.

As with age, we found that more than half of the patients reported difficulty with sleep as the most frequent and distressing psychological symptom. Results further showed that those who reported pain were more likely to experience difficulty sleeping. Among the general population, more than half of individuals reporting chronic pain also report problems with sleep [34]. Failure to treat pain adequately may lead to decreased functional status, mood, and sleep disturbances [35]. Other social factors, such as race, have also been shown to impact sleep habits and patterns among patients experiencing chronic pain. Green and colleagues [36] found that blacks, men, and younger adults reporting chronic pain had a higher prevalence of poor sleep quality. Further studies examining these and other social factors may provide a more comprehensive assessment of the impact pain has on sleep patterns among cancer patients in general and among those from diverse race groups in particular. Knowledge of the nature and prevalence of sleep problems can provide the basis for new approaches to supportive care, as many sleep problems can be effectively treated [37].

The study results stress the importance of psychosocial care and services for cancer patients and their families. One strategy, cognitive-behavioral therapy (CBT), has been effective in improving pain and pain-related problems among cancer patients [38, 39]. Furthermore, tailored CBT approaches to address identified predictors such as age, race, SES, and behaviors, can result in greater improvements of the cancer patient’s pain experience [39, 40]. More research is needed as CBT trials among cancer survivors are limited. Thus, CBT and other evidence-based strategies need to be better understood and practiced within a multidisciplinary team involving oncologists, nurses, social workers, physical and occupational therapists, psychologists, psychiatrists, etc. in order address the biopsychosocial needs of the cancer patient and their families.

Although this study demonstrated pain and specific psychological distress-related symptoms, there were some limitations that must be acknowledged. First, the cross-sectional design of the study does not allow for an analysis of reported relationships over time, particularly as we focus on indicators of pain, sleep and psychological distress. Future studies can benefit in using a longitudinal design to examine the temporal relationship of these study variables. Second, because the study participants were primarily White, with at least a high school education, the study results cannot be generalized to other race (or socioeconomic status) populations. Additionally, the criteria for study participation was not limited to a specific cancer diagnosis, prognosis, or treatment regimen, therefore we cannot definitively compare these findings to other studies examining specific cancer diagnoses. Similarly, while all the patients were diagnosed with cancer, the pain associated with the disease was not discernible between that of cancer and/or of another chronic medical illness (although more than half reported their pain was due to cancer).

## Conclusions

Results from this study add to the limited research exploring how specific distress-related symptoms influence reports of pain (frequency, presence, distress-related) in adult cancer patients. Future research on psychological distress and other social indicators, such as satisfaction with pain management/treatment, race, sex, and socioeconomic status are needed. Specifically, assessing the influence of satisfaction with pain treatment may yield needed information for health care professionals to better understand the level of distress, which may impact treatment adherence and/or seeking medical care. Establishing this knowledge-base can inform education for health care providers, while providing a quality of improvement for systems that provide care to patients with cancer.

Further assessment of identified predictors such as age, race or ethnicity, socioeconomic status, and other physical, behavioral and social indicators may similarly allow for a comprehensive understanding of the daily challenges and concerns of the cancer patient, while providing the resources for nurses, clinicians, and researchers to better meet the needs of this patient population.

## Abbreviations

BPS: Biopsychosocial
ECOG-PS: Eastern cooperative oncology group performance status
IOM: Institute of medicine
MSAS: Memorial symptom assessment scale
NCI: National cancer institute
PHYS: Physical symptoms
PSYCH: Psychological

## References

[1] Patrick D, Ferketich SL, Fram PS (2004). National Institutes of Health state of the science conference statement: symptom management in cancer: pain, depression and fatigue. July 15–17, 2002. J Natl Cancer Inst Monogr.

[2] Harrison JD, Young JM, Price MA, Butow PN, Solomon MJ (2009). What are the unmet supportive care needs of people with cancer? A systematic review. Support Care Cancer.

[3] Linden W, Vodermaier A, MacKenzie R, Greif D (2012). Anxiety and depression after cancer diagnosis: Prevalence rates by cancer type, gender, and age. J Affect Disord.

[4] Lowery AE, Greenberg MA, Foster SL, Clark K, Casden DR, Loscalzo M, Bardwell WA (2012). Validation of a needs-based biospychosocial distress instrument for cancer patients. Psychooncology.

[5] Kirkova J, Walsh D, Rybicki L (2010). Symptom frequency and distress in advanced cancer. Palliat Med.

[6] Ryan D, Gallagher P, Wright S, Cassidy E (2011). Methodological challenges in researching psychological distress and psychiatric morbidity among patients with advanced cancer: What does the literature (not) tell us?. Palliat Med.

[7] Kasparian NA, Sansom-Daly U, McDonald RP, Meiser B, Butow PN, Mann GJ (2012). The nature and structure of psychological distress in people at high risk for melanoma: a factor analytic study. Psycho-Oncology.

[8] Mertz BG, Bistrup PE, Johansen C, Dalton SO, Deltour I, Kehlet H, Kroman N (2012). Psychological distress among women with newly diagnosed breast cancer. Eur J Oncol Nurs.

[9] Poe JK, Hayslip JW, Studts JL (2012). Decision making and distress among individuals diagnosed with follicular lymphoma. J Psychosoc Oncol.

[10] Krok J, Baker TA, McMillan SC (2013). Age differences in the presence of pain and psychological distress in younger and older cancer patients. J Hosp Palliat Nurs.

[11] Shen Johnson M, Redd WH, Winkel G, Badr H (2014). Associations among pain, pain attitudes, and pain behaviors in patients with metastatic breast cancer. J Behav Med.

[12] Nishiura M, Tamura A, Nagai H, Matsushima E (2015). Assessment of sleep disturbance in lung cancer patients: relationship between sleep disturbance and pain, fatigue, quality of life and psychological distress. Palliat Support Care.

[13] Zara C, Baine N (2002). Cancer pain and psychosocial factors: A critical review of the literature. J Pain Symptom Manage.

[14] Mystakidou K, Tsilika E, Parpa E, Katsouda E, Galanos A, Vlahos L (2006). Psychological distress of patients with advanced cancer. Cancer Nurs.

[15] Arraras JL, Wright SJ, Jusue G, Tejedor M, Calvo JI (2002). Coping style, locus of control, psychological distress and pain-related behaviours in cancer and other diseases. Psychol Health Med.

[16] Linden W, Girgis A (2012). Psychological treatment outcomes for cancer patients: what do meta-analyses tell us about distress reduction?. Psychooncology.

[17] Baker TA, Green CR (2005). Intrarace differences among Black and White Americans presenting for chronic pain management: The influence of age, physical health, and psychosocial factors. Pain Med.

[18] Krok JL, Baker TA, McMillan S (2013). Age differences in the presence of pain and psychological distress in younger and older cancer patients. J Hosp Palliat Nurs.

[19] Ashing-Giwa KT, Padilla G, Tejero J (2004). Understanding the breast cancer experience of women: A qualitative study of African American, Asian American, Latina and Caucasian cancer survivors. Psychooncology.

[20] Krok-Schoen J, Baker TA. Gender differences in personality and cancer-related pain among older cancer patients. Journal of Gender Studies. 2015. (E-published ahead of print).

[21] Hadijstavropoulos T, Craig KD, Duck S, Cano A, Goubert L, Jackson PL. Fitzgerald TD.A biopsychosocial formulation of pain communication. Psychol Bull. 10.1037/a0023876 21639605

[22] Portenoy RK, Thaler HT, Kornblith AB (1994). The Memorial Symptom Assessment Scale: an instrument for the evaluation of symptom prevalence, characteristics and distress. Eur J Cancer.

[23] McMillan SC, Tofthagen C, Morgan MA (2008). Relationships among pain, sleep disturbances, and depressive symptoms in outpatients from a comprehensive cancer center. Oncol Nurs Forum.

[24] McMillan SC, Small BJ, Haley WE (2011). Improving hospice outcomes through systematic assessment: A clinical trial. Cancer Nurs.

[25] Dekker J, Boot B, van der Woude LHV, Bijlsma JWJ (1992). Pain and Disability in Osteoarthritis: A Review of Biobehavioral Mechanisms. J Behav Med.

[26] Oken MM, Creech RH, Tormey DC, Horton J, David TE, McFadden ET (1982). Toxicity and response criteria of the Eastern Cooperative Oncology Group. Am J Clin Oncol.

[27] Lynch BM, Cerin E, Owen N, Aitken JF (2007). Associations of leisure-time physical activity with quality of life in a large, population-based sample of colorectal cancer survivors. Cancer Causes Control.

[28] Trinh L, Plotnikoff RC, Rhodes RE, North S, Courneya KS (2011). Associations of leisure-time physical activity with quality of life in a large, population-based sample of colorectal cancer survivors. Cancer Epi Biomarkers Prevent.

[29] Wiggins MS, Simonavice EM (2010). Cancer prevention, aerobic capacity, and physical functioning in survivors related to physical activity: A recent review. Cancer Manag Res.

[30] Gagliese L, Jovellanos M, Zimmermann C, Shobbrook C, Warr D, Rodin G (2009). Age-related patterns of adaption to cancer pain: A mixed method study. Pain Med.

[31] Lo C, Lin J, Gagliese L, Zimmermann C, Mikulincer M, Rodin G (2010). Age and depression in patients with metastatic cancer: The protective effects of attachment security and spiritual wellbeing. Ageing Soc.

[32] Politi MC, Enright TM, Weihs KL (2007). The effects of age and emotional acceptance on distress among breast cancer patients. Support Care Cancer.

[33] Davison SN, Jhangri GS (2005). The impact of chronic pain on depression, sleep, and the desire to withdraw from dialysis in hemodialysis patients. J Pain Symptom Manage.

[34] Pilowsky L, Crettenden I, Townley M (1985). Sleep disturbance in pain clinic patients. Pain.

[35] Green CR, Nadao-Brumblay SK, Hart-Johnson T (2009). Sleep problems in a racially diverse chronic pain population. Clin J Pain.

[36] Morris BA, Thorndike FP, Ritterband LM, Glozier N, Dunn J, Chambers SK (2015). Sleep disturbance in cancer patients and caregivers who contact telephone-based help services. Support Care Cancer.

[37] Baker TA, Whitfield KE (2015). Intrarace group variability in characteristics of self-reported pain and sleep difficulty in older African Americans with arthritis. J Transcult Nurs..

[38] Tatrow K, Montegomery GH (2006). Cognitive behavioral therapy techniques for distress and pain in breast cancer patients: a meta-analysis. J Behav Med.

[39] Syrjala KL, Jensen MP, Mendoza ME, Jean CY, Fisher HM, Keefe FJ (2014). Psychological and behavioral approaches to cancer pain management. J Clin Oncol.

[40] Dalton JA, Keefe FJ, Carlson J, Youngblood R (2004). Tailoring cognitive-behavioral treatment for cancer pain. Pain Manage Nurs.

